# Threshold shifts and developmental temperature impact trade-offs between tolerance and plasticity

**DOI:** 10.1098/rspb.2023.2700

**Published:** 2024-02-07

**Authors:** Belinda van Heerwaarden, Carla Sgrò, Vanessa M. Kellermann

**Affiliations:** ^1^ School of BioSciences, The University of Melbourne, Parkville 3010 Victoria, Australia; ^2^ School of Biological Sciences, Monash University, Clayton 3800, Victoria, Australia; ^3^ School of Agriculture Biomedicine and Environment, La Trobe University, Bundoora 3086, Victoria, Australia

**Keywords:** CT_MAX_, thermal, acclimation, hardening, adaptive, climate

## Abstract

Mounting evidence suggests that ectotherms are already living close to their upper physiological thermal limits. Phenotypic plasticity has been proposed to reduce the impact of climate change in the short-term providing time for adaptation, but the tolerance-plasticity trade-off hypothesis predicts organisms with higher tolerance have lower plasticity. Empirical evidence is mixed, which may be driven by methodological issues such as statistical artefacts, nonlinear reaction norms, threshold shifts or selection. Here, we examine whether threshold shifts (organisms with higher tolerance require stronger treatments to induce maximum plastic responses) influence tolerance-plasticity trade-offs in hardening capacity for desiccation tolerance and critical thermal maximum (CT_MAX_) across *Drosophila* species with varying distributions/sensitivity to desiccation/heat stress. We found evidence for threshold shifts in both traits; species with higher heat/desiccation tolerance required longer hardening treatments to induce maximum hardening responses. Species with higher heat tolerance also showed reductions in hardening capacity at higher developmental acclimation temperatures. Trade-off patterns differed depending on the hardening treatment used and the developmental temperature flies were exposed to. Based on these findings, studies that do not consider threshold shifts, or that estimate plasticity under a narrow set of environments, will have a limited ability to assess trade-off patterns and differences in plasticity across species/populations more broadly.

## 1. Introduction

Predicted rises in temperatures and changes in precipitation [1,2] are likely to impact the distribution, abundance and extinction risk of many species [3–5]. Comparisons of upper thermal tolerance across ectotherms suggest that many species may already live close to their upper physiological thermal limits [6–10]. Although some species can show rapid adaptation to climate change [11–13], studies also suggest that genetic adaptation to increase upper thermal limits and desiccation tolerance may be limited, especially in tropical species [8,14–16]. Because phenotypic plasticity can increase tolerance to environmental stresses like heat, cold and desiccation after short-term exposure (minutes/hours) to sub-lethal temperatures/humidity levels (hardening) or longer-term exposure (days/weeks) to warmer temperatures (acclimation) [17], it has been proposed that plasticity may reduce the impact of climate change in the short-term providing time for adaptation [18–21]. Nonetheless, predicting whether phenotypic plasticity will indeed buffer species from climate change requires understanding what drives and limits the evolution of both tolerance and plasticity.

The tolerance-plasticity trade-off hypothesis predicts that plasticity will be lower in individuals with higher baseline (inherent/basal) tolerance [22–25]. Thus, species adapted to warmer/drier environments may be more vulnerable to climate change than predicted because their current tolerance is already close to current maximum habitat temperatures and humidity limits, and they have a limited ability to increase their tolerance via plasticity [22]. Most studies have examined tolerance-plasticity trade-offs by looking for negative associations between tolerance and plasticity across different populations or species [23–26]. The results of these studies are mixed; some studies have found trade-off patterns, while others have found no association, or the opposite pattern, where populations/species with the lowest baseline tolerance also have the lowest plasticity (positive association), making these populations/species more vulnerable to climatic extremes [23,25,27]. Although most attention has focused on heat tolerance [24], tolerance-plasticity trade-offs may also impact the evolution of tolerance and plasticity in other stress traits (e.g. desiccation [28], cold [29–31], salinity [32], CO_2_ [33] and herbivore tolerance [34]).

While trade-off patterns suggest a constraint to evolving both high tolerance and plasticity [35], associations detected across populations, or species, may occur because of methodological issues in the way we estimate plasticity [24,36]. Statistical artefacts may arise when plasticity is estimated using only two temperatures or treatments (e.g. hardening). This is because tolerance estimated using one of these temperatures/treatments will be included in both the estimate of plasticity *and* baseline tolerance, which will potentially be confounded by statistical non-independence between the response and predictor variable or regression to the mean [37–40]. A recent study has shown that this statistical artefact can be substantial [40], suggesting that studies need to consider this issue when designing experiments to assess the trade-off tolerance-plasticity hypothesis.

Even in studies where statistical bias is not an issue, associations between tolerance and plasticity may also arise (or be missed) if plasticity is underestimated in species with different levels of tolerance because of the temperatures/treatments that are used to estimate plasticity. Trade-off patterns may occur if plasticity is underestimated in species/populations/lines with high tolerance if more intense, or longer hardening treatments are required to induce hardening responses because shorter/less extreme treatments are not stressful enough to trigger their hardening response (threshold plasticity hypothesis [24,41]). Threshold shifts could also be influenced by rearing temperatures/thermal history, if tolerance changes, due to developmental acclimation. For example, hardening capacity (HC) may be lower at higher temperatures if tolerance and the thermal threshold for heat hardening increases [42]. There is some evidence at the molecular genetic level that higher temperatures may be needed to induce the heat shock response in species adapted to warmer habitat temperatures, in line with the threshold shift hypothesis [42–45]. For instance, thermal thresholds for the transcriptional activation of the heat shock response (e.g. heat shock proteins (HSP) and heat shock transcription factor) have been found to vary seasonally and across species according to habitat temperatures (reviewed in [44,45]), implicating a ‘cellular thermometer’ for the heat shock response and plasticity [43]. However, no studies to our knowledge have explicitly tested whether species with high tolerance, or adapted to warmer habitats, require more intense hardening treatments to induce the maximum hardening response. If thresholds influence HC more broadly, trade-off patterns could emerge because the treatments used to induce a response are not stressful enough for more tolerant individuals/populations/species, or due to thermal history, rather than evolutionary or functional constraints limiting the evolution of tolerance and plasticity.

In three published papers [26,46,47], we found some evidence for changes in HC across different temperatures/treatments for heat/desiccation tolerance in *Drosophila,* but did not explicitly explore whether threshold shifts may be influencing hardening responses and trade-off patterns. In [47], we found HC decreased at higher developmental acclimation temperatures in a tropical and temperature population of *D. melanogaster.* In [46], we focused on species differences in maximum HC for critical thermal maximum (CT_MAX_) at different developmental temperatures, but did not consider the data on different hardening durations across the 10 *Drosophila* species. Further, this paper did not explore tolerance-plasticity trade-offs. In [26], tolerance-plasticity trade-off patterns across 32 different *Drosophila* species were found using a common (3.5 h) hardening treatment, but threshold shifts and tolerance-trade-off patterns using the treatment that induced the unadjusted maximum hardening response were not considered. Using data from [26] and [46], here, we examined whether threshold shifts are evident for desiccation and heat tolerance, whether the decreases in HC at higher developmental temperatures we found in *D. melanogaster* [47] occur in other species, and whether threshold shifts and developmental temperature influence trade-off patterns between baseline tolerance and plasticity.

## 2. Material and methods

### (a) Sample information

For CT_MAX_, 10 *Drosophila* species—varying in their distribution and heat tolerance—were collected from the field between 2013 and 2015, with experiments completed in 2015–2016 (see [46,47] for further details). For desiccation, 32 *Drosophila* species were collected from three different sources: 21 were collected from the field in Australia between 2010 and 2015, with experiments completed in 2013–2016, eight were obtained from stock centres and three species were sourced from long-term laboratory stocks in Denmark, with experiments completed in 2015 [26]. While some of the samples were taken from long-term laboratory colonies, so laboratory adaptation cannot be excluded [48], half of the species were assessed for heat tolerance within 4 months of field collection and 20% of flies for the desiccation experiment were assessed within a year of collection. Furthermore, [26] found no association between time in the laboratory and HC or desiccation tolerance.

### (b) CT_MAX_ experimental details

Flies were reared from egg to adults across four fluctuating temperatures: mean 20°C (15–25°C), 23°C (18–28°C), 26°C (21–31°C) and 28°C (23–33°C) (see [46] and electronic supplementary material, figure S1 for detailed experimental set-up). Because different species may require different hardening treatments to induce hardening responses (threshold plasticity hypothesis), we exposed each species to several different hardening treatments (electronic supplementary material, figure S1). Although altering the hardening temperature and time would have been optimal, because of the large number of possible combinations, we decided to alter the exposure period of heat stress (15 min, 30 min, 60 min and 90 min) and keep the hardening temperature constant (37°C). Any hardening treatments that induced mortality were excluded from the analysis to avoid selection. On completion of the hardening treatment, flies were given a recovery period of 23 h at 25°C [46]. Flies were then placed into individual vials and put into a water bath that was slowly ramped up from 25°C at a rate of 0.1°C per minute to estimate CT_MAX_ [49]. Control flies, not pre-exposed to a heat stress, were also included in the estimation of CT_MAX_ (unhardened CT_MAX_). The time and temperature at which flies no longer moved and succumbed to heat stress was scored.

### (c) Desiccation experimental details

Desiccation hardening responses were induced by exposing approximately 100 female flies (10 per vial) to a non-lethal desiccation stress of 5–10% relative humidity (RH) for varying time periods (see [26] and electronic supplementary material, figures S2 and S3) for more details on hardening treatments). Hardening treatments included a common treatment of 3.5 h for all species (which did not result in mortality in any species except *D. bipectinata* and *D. equinoxialis,* where only a 2 h pre-treatment was used). Following hardening, flies were placed into vials containing food (90–100% humidity) for a 9 h recovery period at 25°C, prior to being assessed for desiccation resistance at 5–10% RH (see below). Control flies (unhardened) were set up in a similar manner, except that they were maintained on food (90–100% humidity) during the hardening pre-treatment. Desiccation resistance was scored every hour until 50% mortality was observed in each vial, with each vial providing a data point which was analysed as LT50 [26].

### (d) Analysis

#### (i) Hardening capacity for CT_MAX_

HC for CT_MAX_ was calculated by subtracting average unhardened CT_MAX_ from individual estimates of hardened CT_MAX_ for each species. HC was calculated for each hardening treatment (i.e. 15, 30 min etc.) at each developmental acclimation temperature (e.g. HC_20_ was calculated as individual hardened CT_MAX_ at 20°C – species' average unhardened CT_MAX_ at 20°C).

#### (ii) Hardening capacity for desiccation tolerance

HC for desiccation tolerance was calculated for each hardening time by subtracting average unhardened desiccation LT50 estimates from hardened desiccation LT50 estimates for each hardening duration, for each species. For two species (*D. bipectinata* and *D. equinoxialis*), a 3.5 h desiccation stress induced mortality, so HC was only assessed using a 2 h stress treatment.

#### (iii) Exploring whether hardening time and acclimation temperature influence hardening capacity

*Phylogenetic framework and environmental data.* We analysed both the heat and desiccation tolerance data within a phylogenetic framework using the phylogenetic least squares method in the caper package in R [50]. Phylogenies for heat and desiccation resistance were taken from [46] for heat and [26] for desiccation. For the heat data, preliminary analyses found no phylogenetic signal in the relationship between hardening time and baseline tolerance/climate, or between baseline tolerance and HC. Furthermore, the heat data represented a small number of species, and arguably an insufficient number to capture meaningful phylogenetic signal [51]. For these reasons, we present all the results for heat from standard linear models (which is the default in caper when there is no phylogenetic signal). Given the larger number of species for desiccation resistance, we present the phylogenetic analyses for this trait.

To examine relationships between HC, time to harden, baseline tolerance and the environment we chose to focus on two environmental variables (annual precipitation (*P*_ANN_) and maximum temperature of the warmest month (*T*_MAX_)). These environmental variables have been linked to desiccation and heat tolerance and their plasticity across different *Drosophila* species [8,26,46,52]. Briefly, environmental data was extracted from WorldClim (https://www.worldclim.org) based on the average distribution of each species (see [46] for detailed information on how data on these climatic variables were extracted).

CT_MAX_*.* We first examined whether HC changed across different developmental acclimation temperatures/hardening treatments across species. We used general linear models (lme4 v.1.1–28 [53]) and ANOVAs (car 3.1–12 [54]) in R (version 1.3.959) to model and look at the effect of species, developmental temperature and hardening time (all fixed effects) on HC for CT_MAX_.

To investigate whether shifting thresholds influenced hardening responses, we examined whether species with higher heat tolerance/developing at warmer temperatures required longer hardening treatments to induce the largest hardening response. For each species, we first assessed which hardening duration resulted in the highest hardening response (the difference between unhardened and hardened CT_MAX_) at each temperature. Using the hardening duration which induced the maximum/highest hardening response as our response variable, we used linear regression models in R to examine whether there was an association between the predictor baseline CT_MAX_ and the response variable hardening duration required to achieve the highest hardening response (averaged across all developmental temperatures) across species overall, or between hardening duration and developmental temperature in species individually. Next, we used a multiple regression model in R to explore whether there was an association between the response variable time required to achieve the maximum heat hardening response and the predictor environmental variables *P*_ANN_ and *T*_MAX_. We then used the car package in R [54] to create added-variable plots for this multiple regression model.

*Desiccation tolerance.* Similar to CT_MAX_, we first used general linear models and ANOVAs in R [53,54] to look at the effect of species and hardening time (both fixed effects) on HC for desiccation tolerance. We then assessed which hardening duration resulted in the highest hardening response for each species.

Using phylogenetic linear models [50], we first examined whether there was an association between the predictor variable baseline desiccation tolerance and the response variable hardening time required to achieve the largest hardening response across species. Next, we determined whether the response variable time required to achieve the maximum desiccation hardening response was associated with the predictor variable *P*_ANN_.

#### (iv) Changes in hardening capacity for CT_MAX_ across developmental temperature

Given that our previous findings in *D. melanogaster* suggested that plasticity may decrease at increasing developmental temperatures [47], we investigated whether plasticity decreases at increasing developmental temperature in *D. melanogaster* when accounting for threshold shifts (i.e. using different hardening treatments until mortality occurs), as well as in other species. To explore this, we first used linear regression models in R to look at the association between the predictor variable developmental temperature and the response variable HC (using the hardening treatment that induced the largest response) estimated at each developmental temperature (e.g. HC_20_) for each species individually. We then used linear regression to explore whether there was an association between the response variable HC and predictor variable developmental temperature across species.

To assess whether changes in HC across development acclimation temperature could be predicted by a species’ CT_MAX_, we used linear regression to examine the relationship between the predictor variable average unhardened CT_MAX_, and the response variable HC_MAX_ temperature slope, which used the slope of linear regression between an individual species' HC and developmental temperature (calculated above).

#### (v) Exploring whether threshold shifts or developmental temperature influence associations between tolerance and plasticity

CT_MAX*.*_ We first explored whether there was evidence for an association between baseline CT_MAX_ and HC across the 10 species, and whether associations between tolerance and HC differed depended on whether we used a common hardening treatment all species could endure without mortality, or the time that induced the maximum hardening response for each species. Because unhardened tolerance estimates and plasticity calculated using the difference between hardened and unhardened tolerance at individual developmental acclimation temperatures are not statistically independent (because baseline CT_MAX_ at 20°C is one of the variables used directly in the comparison *and* in the estimation of HC (discussed in [24])), we used unhardened CT_MAX_ averaged across *all* temperature treatments as our estimate of ‘baseline’ CT_MAX_ to explore trade-off patterns with HC. We used linear regression models to investigate the association between the predictor variable baseline CT_MAX_ and the response variable HC in flies developing at fluctuating 20°C estimated using a common hardening treatment (15 min at 37°C: HC_15_). We then repeated this analysis using the hardening treatment that induced the highest hardening response in each species (HC_MAX_) to re-examine the relationship between baseline CT_MAX_ and HC_MAX_ at 20°C.

Because HC may change across developmental temperature, we were also interested in exploring whether the association between average baseline CT_MAX_ and HC_MAX_ changed when HC was estimated at warmer developmental acclimation temperatures (e.g. 23, 26 and 28°C). We then used linear regression models to look at associations between the predictor variable average baseline CT_MAX_ and the response variable HC_MAX_ estimated on flies developing at 23, 26 and 28°C. We compared whether the regression slopes changed significantly when HC was estimated at different developmental acclimation temperatures by calculating the Z statistic, estimated by taking the difference between the two coefficients from the individual linear models, and then dividing this by a pooled standard error [55]. This was done for each pair of linear regression models comparing the association between tolerance and HC estimated at each of the four developmental acclimation temperatures.

*Desiccation tolerance.* Because unhardened desiccation tolerance is directly used in the calculation of HC, (so is not statistically independent), we explored how baseline resistance and plasticity trade-off along an environmental gradient [22,26,47]. If associations between precipitation and tolerance/plasticity show opposing patterns, it would indicate a trade-off (as species adapted to lower levels of precipitation have higher tolerance, but low plasticity), while parallel associations would indicate species adapted to lower precipitation have both higher tolerance and higher plasticity. Kellermann & Sgro [46] found opposing associations between precipitation and tolerance/HC using a common treatment that did not cause mortality in any species, suggesting species adapted to lower levels of precipitation had higher tolerance, but lower HC. We used phylogenetic linear models [50], using desiccation tolerance/HC as the response variable and *P*_ANN_ as the predictor variable, to assess whether the association between *P*_ANN_ and HC differed when estimated using this common hardening treatment (3.5 h: HC_3.5_) and a hardening treatment that induced the highest hardening response (HC_MAX_).

## 3. Results

### (a) Is there evidence for threshold shifts?

#### (i) CT_MAX_

Given the prediction that longer hardening durations may be required to induce hardening responses in species with higher heat tolerance (threshold plasticity hypothesis [24]), we first explored the effect of hardening time on HC across different species. Since tolerance can increase at warmer developmental temperatures (due to acclimation), we also looked at whether longer hardening durations were required to induce the maximum hardening response at warmer temperatures during development.

**Figure 1.**
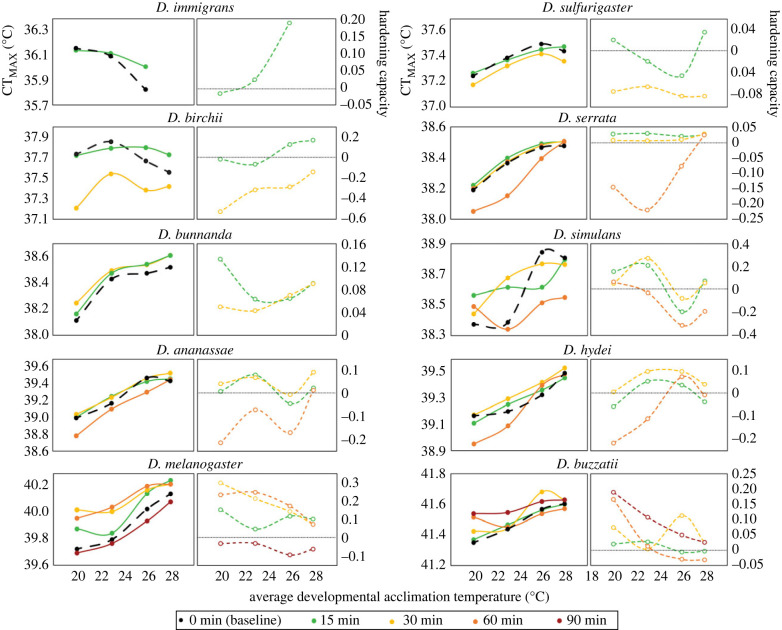
Hardening responses can be influenced by hardening duration and developmental acclimation temperature. CT_MAX_ and hardening capacity for CT_MAX_ for individual species at different fluctuating developmental acclimation temperatures when exposed to a hardening treatment of 37°C for different hardening durations (see colour key), 23 h prior to assessing CT_MAX_. Baseline (unhardened) CT_MAX_ is shown as the large black circles and dashed lines. Each circle is the mean CT_MAX_ (solid) or hardening capacity (open) at each developmental temperature. The dotted black lines indicate zero hardening capacity.

HC differed across species and hardening time but did not vary significantly across developmental temperature (electronic supplementary material, table S1 and figure 1). We found a significant interaction between species and hardening time (electronic supplementary material, table S1), indicating that hardening time influenced hardening capacity differently across species (figure 1). Hardening capacity in some species, particularly those with high heat tolerance (e.g. *D. melanogaster* and *D. buzzatii*), was higher when they were exposed to hardening treatments of 1 hour or more, while other more sensitive species showed higher hardening capacity under shorter hardening durations (e.g. *D. sulfurigaster, D. birchii* and *D. serrata*), with longer hardening durations decreasing, rather than increasing, heat tolerance (figure 1).

**Figure 2.**
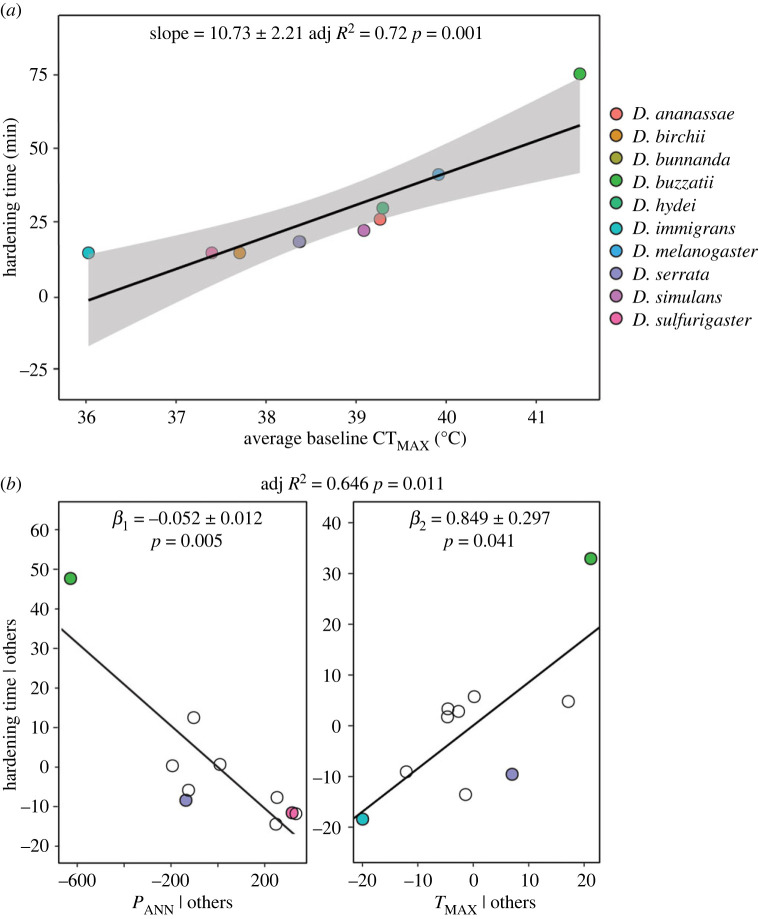
Testing the threshold hypothesis for the induction of plastic responses for heat tolerance. (*a*) The relationship between the time to achieve the maximum heat hardening response and average baseline (unhardened) CT_MAX_ for each species (see colour key). Statistics and solid lines are from the fitted linear model and the shaded areas are the 95% confidence intervals. (*b*) Added variable plots displaying the relationship between the time taken to achieve the maximum hardening response for CT_MAX_ for each species and each predictor variable (annual precipitation (*P*_ANN_), maximum habitat temperature (*T*_MAX_)) in the multiple linear regression model, while holding the value of all other predictor variables constant. The coloured points in each plot in (*b*) represent the observations with the largest residuals and the observations with the largest partial leverage (refer to colour legend for species).

In line with expectations under the threshold shift hypothesis, we found a significant positive relationship between baseline CT_MAX_ and the time to elicit the highest hardening response across species, with the species with the highest heat tolerance generally requiring longer hardening treatments (figure 2*a*). We also found the hardening time required to achieve the maximum hardening response was associated with environmental variables commonly found to explain variation in CT_MAX_ across different *Drosophila* species [8,46]; species from drier and hotter habitats needed longer heat hardening treatments to achieve their maximum hardening response (figure 2*b*).

We did not detect a significant interaction between developmental temperature and hardening time, indicating that hardening time did not generally influence hardening capacity at different developmental temperatures (figure 1; electronic supplementary material, table S1). Thus, while we saw changes in plasticity across developmental temperatures (explored below), these changes in hardening capacity were probably not driven by threshold shifts, as species did not need longer hardening treatments to induce the maximum hardening response at warmer developmental temperatures (figure 1; electronic supplementary material, figure S4). We did, however, detect a significant interaction between developmental temperature, hardening time and species (electronic supplementary material, table S1 and figure 1). This was because some species that showed low, and sometime negative, hardening responses at lower developmental temperatures (indicative of physiological damage) showed larger hardening responses at higher developmental temperatures (especially under longer hardening durations) (figure 1). Thus, HC was affected by both developmental temperature and hardening time, but only for some species.

#### (ii) Desiccation tolerance

**Figure 3.**
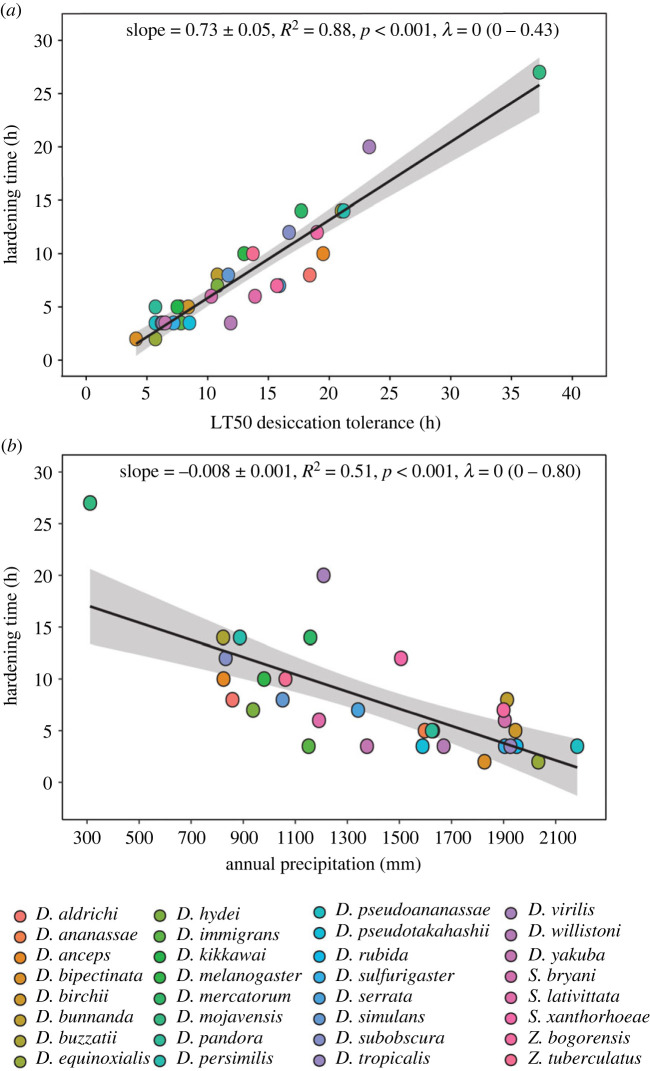
Testing the threshold hypothesis for the induction of plastic responses for desiccation tolerance. The relationship between the time to achieve the maximum hardening response for desiccation tolerance and (*a*) baseline (unhardened) desiccation tolerance, and (*b*) environment (annual precipitation). Each coloured circle represents an individual species (see colour legend). Statistics and solid lines are from the fitted phylogenetic linear model and the shaded areas are the 95% confidence intervals.

Hardening time had a significant effect on hardening capacity (electronic supplementary material, table S1 and figure S3). Although most species showed an increase in hardening capacity with increasing hardening duration and the highest hardening response was generally observed at the highest hardening duration (26/32 species), the hardening time that induced the highest hardening response differed across species (electronic supplementary material, table S1 and figure S3). Similar to CT_MAX_, we found a significant positive linear relationship between baseline (unhardened) desiccation LT50 and the time to elicit the highest hardening response across species (figure 3*a*), indicating that species with higher desiccation tolerance required longer hardening treatments to maximize their hardening response, in line with the threshold shift hypothesis. For some species (especially those with higher baseline desiccation tolerance), the difference in hardening capacity between the 3.5 h treatment and the hardening treatment that induced the maximum hardening response was quite large (electronic supplementary material, figure S3). For example, hardening capacity in *D. virilis* was negative (−1.5 h) using a 3.5 h hardening treatment compared with a 9 h improvement in desiccation tolerance using the treatment that induced the maximum hardening response (20 h hardening treatment) (electronic supplementary material, figure S3). We also found a significant negative association between hardening time to achieve the maximum hardening response and annual precipitation (figure 3*b*), indicating that species occupying drier habitats needed longer desiccation hardening treatments to achieve their maximum hardening response.

### (b) Does maximum hardening capacity for heat tolerance change across developmental temperatures?

**Figure 4.**
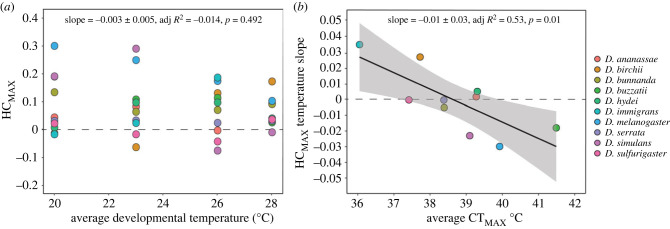
Changes in CT_MAX_ plasticity with increasing developmental acclimation temperature are associated with heat tolerance. (*a*) The relationship between baseline CT_MAX_ and maximum hardening capacity (HC_MAX_) estimated in flies developing under different fluctuating developmental acclimation temperatures; (*b*) the relationship between average baseline CT_MAX_ and HC_MAX_ temperature slope (from electronic supplementary material, figure S5) across developmental temperature in 10 *Drosophila* species. Each coloured circle represents a single species (see colour legend). Statistics and solid lines are from the fitted linear regression model and the shaded areas are the 95% confidence interval of these models.

Even when accounting for threshold shifts, trade-off patterns could change if hardening capacity differs across developmental temperatures. Using the hardening time that elicited the highest hardening response for each species (HC_MAX_, see above), we found no association between hardening capacity and developmental temperature across species (figure 4*a*). When we explored the association between HC_MAX_ and developmental temperature in species individually, we found species with higher heat tolerance (*D. melanogaster* and *D. buzzatii*) were more likely to show declines in HC at warmer temperatures (HC temperature slope, electronic supplementary material, figure S5), while species with lower heat tolerance (*D. birchii* and *D. immigrans*) were more likely to show increases in HC with increasing developmental temperature (electronic supplementary material, figure S5 figure 4*b*).

### (c) Do threshold shifts/changes in plasticity across temperature influence trade-off patterns?

#### (i) CT_MAX_

Because we detected evidence for threshold shifts and found that changes in HC_MAX_ across developmental temperature were linked to species baseline CT_MAX_ (figure 4*b*), we explored whether plasticity-tolerance trade-offs/ positive correlations differed depending on which hardening treatments and developmental temperature ranges were used to estimate plasticity.

**Figure 5.**
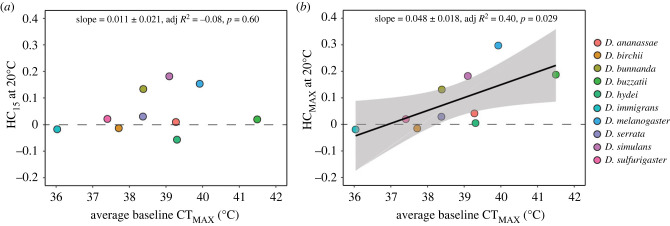
Associations between tolerance and hardening capacity change depending on hardening treatment. The relationship between baseline (unhardened) CT_MAX_ and (*a*) average hardening capacity at 20°C using a common hardening treatment of 37°C for 15 min (HC_15_) and (*b*) hardening capacity at 20°C using the hardening treatment that induced the maximum hardening response (HC_MAX_ across 10 different *Drosophila* species (see colour legend)). Statistics and solid lines are from the fitted linear regression model and the shaded areas are the 95% confidence interval of this model. Each coloured point (see colour legend) represents a single species’ average CT_MAX_ across all developmental temperatures (which reduced the regression to the mean issue as average unhardened CT_MAX_ was not used to calculate hardening capacity at each temperature) and average HC_MAX_/ HC_15_ assessed at the specified average fluctuating developmental acclimation temperature.

We found no association between average baseline CT_MAX_ and hardening capacity using a hardening treatment of 15 min at 37°C (HC_15_) in flies developing at 20°C (figure 5*a*), indicating that species with higher heat tolerance did not have lower or higher hardening capacity when HC was calculated using a common hardening treatment that all species could endure without mortality (HC_15_). However, when we re-examined this relationship using the treatment that induced the highest hardening response for each species (HC_MAX_), we found a significant positive association between HC_MAX_ at 20°C and average baseline CT_MAX_ (figure 5*b*). Thus, once threshold shifts were considered, species with higher heat tolerance had higher hardening capacity, while species with lower heat tolerance had lower hardening capacity. We also found the association between average baseline CT_MAX_ and HC_MAX_ differed depending on the developmental acclimation temperatures of flies assessed for HC (electronic supplementary material, table S2 and figure S6).

#### (ii) Desiccation tolerance

**Figure 6.**
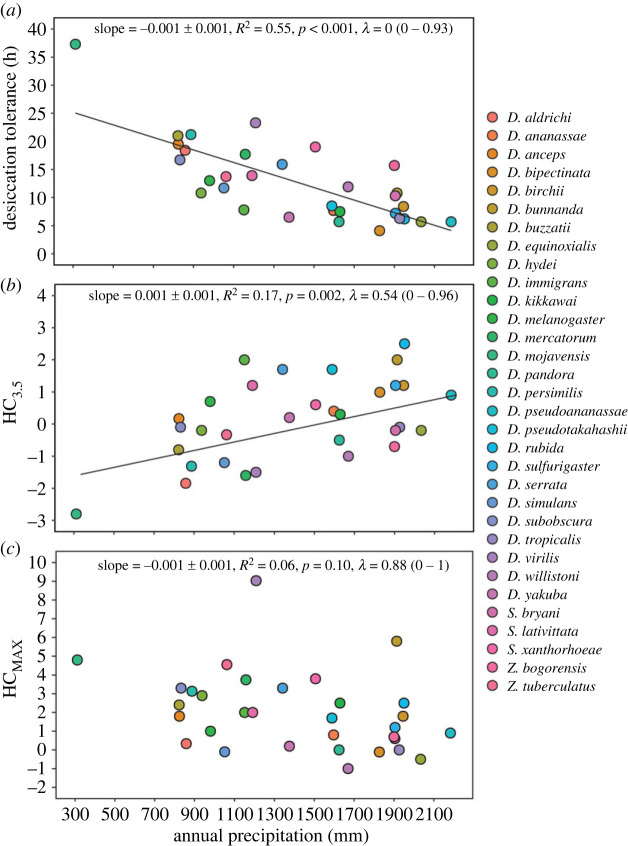
Exploring trade-offs between desiccation tolerance and hardening capacity using different methods. The relationship between annual precipitation and (*a*) baseline desiccation tolerance (LT50), (*b*) hardening capacity using a 3.5 h hardening treatment (HC_3.5_) and (*c*) hardening capacity using the treatment that induced the largest hardening response (HC_MAX_) across 32 *Drosophila* species (see colour legend). Statistics and solid lines are from the fitted linear model.

Kellermann *et al.* [26] found opposing linear associations between annual precipitation and unhardened desiccation tolerance/hardening capacity after 3.5 h pre-treatment (HC_3.5_), suggesting that species adapted to low levels of precipitation had higher desiccation tolerance, but lower hardening capacity, as expected under the tolerance-plasticity trade-off hypothesis. When we explored whether this pattern changed when threshold shifts were considered, by using the maximum hardening response (HC_MAX_), we found that the relationship between environment and desiccation plasticity reversed; species with distributions characterized by low levels of precipitation had both higher desiccation tolerance and higher hardening capacity (albeit the association between annual precipitation and HC_MAX_ was much weaker) (figure 6). These results suggest that trade-offs between desiccation tolerance and hardening capacity across annual precipitation can vary depending on what hardening treatment is used.

## 4. Discussion

Many studies examining associations between tolerance and plasticity have directly compared tolerance and plasticity measured across only two environments [24]. Some recent studies have demonstrated how tolerance-plasticity associations estimated this way may be driven by statistical artefacts [37,38,40]. We explored whether underestimating plasticity in organisms with higher levels of tolerance (threshold shifts) or changes in plasticity across different acclimation temperatures [24,44,47,56] may also influence tolerance-plasticity trade-off patterns.

### (a) Threshold shifts impact plasticity

In line with the threshold shift hypothesis [24,41], we found that longer hardening treatments were required to induce the maximum hardening response in species with higher tolerance for both CT_MAX_ and desiccation tolerance (figures 2*a* and 3*a*). We also found that species adapted to warmer and drier environments required longer hardening treatments to induce the maximum hardening response, suggesting selection might play a role in driving threshold shifts (figures 2*b* and 3*b*). This result was not unexpected, given that thermal thresholds for the transcriptional activation of the heat shock response (e.g. heat shock transcription factor (HSF) and HSPs) can differ among species and have been shown to correlate positively with the level of heat stress encountered in their ecological niche [42,44,45]. However, this is the first study (to our knowledge) to link habitat environmental variables to threshold shifts in plasticity for tolerance across species.

There is also evidence from experimental evolution studies that heat shock response (HSR) induction and plasticity thresholds can evolve [57–59]. Baseline heat tolerance was lower in *D. melanogaster* lines evolving at cooler temperatures, and HSF induction and hardening responses were activated at lower hardening temperatures than lines evolving at warmer temperatures [57,58]. While this suggests that plasticity thresholds can evolve as tolerance increases, longer/hotter hardening treatments may not help when organisms reach an upper physiological limit. For instance, *D. melanogaster* lines evolving at the warmer temperatures in the study above failed to show a hardening response, even under hotter hardening treatments, suggesting that there may be an upper limit to hardening responses [57]. Similar upper limits may also exist for desiccation tolerance and plasticity. *Drosophila melanogaster* lines selected for increased desiccation tolerance had lower hardening capacity compared with unselected lines, which was also not driven by threshold shifts, as longer hardening treatments failed to increase the hardening response in selected lines [28]. Expression studies have also found differences in heat shock responses in selection lines and acclimation/hardening capacities in *Drosophila* species/populations with different levels of thermal tolerance [60–62].

We also hypothesized that longer hardening durations may be required to induce maximum hardening responses at higher developmental acclimation temperatures, as heat tolerance can increase with development acclimation [25,47,63], and thermal thresholds for the transcriptional activation of the heat shock response can vary seasonally and with acclimation temperature in the laboratory in other species [42,44,45,56,64]. We found no evidence that longer hardening durations increased hardening responses at higher developmental acclimation temperatures in any species (figure 1; electronic supplementary material, figure S4). While it is possible that higher hardening temperatures, rather than longer hardening treatments (as explored here), or different recovery periods, may have increased hardening responses at warmer acclimation temperatures, these results suggest that hardening duration thresholds observed across species with different heat tolerance do not apply to changes in heat tolerance through developmental acclimation, at least in these species.

### (b) Decreases in plasticity at higher temperatures depend on baseline heat tolerance

In addition to increases in average temperatures, species will experience frequent and more extreme maximum habitat temperatures [1,2]. In a previous paper, we found that hardening capacity decreased at warmer developmental temperatures in a tropical and temperate population of *D. melanogaster* [47], suggesting a limited capacity for plasticity to buffer temperature extremes during heatwaves. If this pattern is common in other species, plasticity may be more limited than currently predicted [25,65–67].

We found that hardening capacity for CT_MAX_ did not generally decrease at higher developmental acclimation temperatures (figure 4*a*). Instead, we found that changes in hardening capacity across developmental acclimation temperature were again influenced by species' heat tolerance; hardening capacity was more likely to decrease at higher developmental temperatures in species with higher heat tolerance, while species with lower heat tolerance showed increases in hardening capacity at higher developmental temperatures (figure 4*b*). However, in the species with low tolerance, plasticity was higher at warmer developmental temperatures due to a decline in baseline heat tolerance rather than an increase in hardened heat tolerance (figure 1). A decline in baseline thermal tolerance at higher temperatures in less tolerant species may occur because these temperatures are more stressful for species with low tolerance and induces physiological damage, rather than beneficial acclimation [24,68]. It is also possible that this pattern is driven by the range of developmental acclimation temperatures chosen (some of which may have been more stressful for some species than others).

Although further experiments are required to elucidate what factors may be driving decreases in hardening capacity at warmer acclimation temperatures in the species with high tolerance, as discussed above, it is possible that warmer hardening temperatures (rather than longer hardening treatments) may have been required to induce the maximum hardening response at warmer developmental temperatures in species with high tolerance. Species with high tolerance may also have reached their upper physiological limit [28,57]. Changes in hardening capacity across temperatures could also be driven by a mechanistic link between developmental acclimation and reversible hardening capacity, or different costs under varying levels of environmental predictability [69,70]. For example, in organisms from environments with high within-season variability, reversible hardening may evolve to be higher at lower acclimation temperatures to compensate for environmental mismatches between developing and adult life stages [69]. Finally, although we have focused on trade-offs between ‘baseline’ tolerance and hardening capacity, this may not be the only constraint that might limit the evolution of plastic responses [35,71–73].

### (c) Threshold shifts and developmental temperature influence tolerance-plasticity trade-offs/correlations

Associations between hardening treatment, tolerance and environment—for both heat and desiccation tolerance—indicate that studies that use the same hardening treatment across different species with different levels of tolerance and distributions are likely to underestimate plasticity in tolerant individuals, which may influence trade-off patterns. As predicted, we found that threshold shifts altered the conclusions for whether a trade-off or positive association between tolerance and plasticity was detected. Once we took threshold shifts into account, we found no evidence for a trade-off between tolerance and plasticity across species for desiccation tolerance (figure 6). We also found a significant positive association between tolerance and plasticity for CT_MAX_ at 20°C that was not evident when we used a common hardening treatment, or at other developmental acclimation temperatures (figure 5; electronic supplementary material, figure S6).

If acclimation temperature significantly influences hardening capacity, and these effects depend on tolerance (as we found here), then species collected from warmer habitats and acclimated in the laboratory may appear to have less plasticity because of thermal history, rather than because they have lower plasticity [67,74–77]. Furthermore, if threshold shifts or interactions between developmental acclimation and hardening capacity are common, then studies that use only two acclimation temperatures, or use the same hardening treatment for all samples, may detect tolerance-trade-off patterns that are underpinned by these methodological issues rather than evolutionary or physiological constraints [24]. While we have shown how threshold shifts and developmental acclimation can directly influence tolerance-plasticity trade-off patterns, the direction and magnitude of trade-offs across other traits can also change across different environments [78,79], highlighting the importance of considering environmental effects on trade-off patterns generally. Finally, given that past studies have also linked habitat temperatures to HSP induction responses across different populations [42,64], future work should also consider threshold shifts when estimating plasticity across populations, or lines selected for higher tolerance.

#### (i) Conclusion

Most studies investigating tolerance-plasticity trade-offs/correlations use the same hardening treatment for all samples, or estimate plasticity using only two acclimation temperatures [24]. If statistical artefacts, threshold shifts and developmental temperature effects (thermal history) are common, as we have seen for desiccation and CT_MAX_ here, and in [37–40], many previous trade-off studies may also be affected by these issues. This may, in part, explain the equivocal empirical patterns observed across studies [24]. Given these potential problems, it remains unclear whether a general relationship between plasticity and tolerance exists across taxa, although [40] suggests that many studies do indeed suffer from statistical artefacts. Here, we show that even when trade-offs are still evident after statistical artefacts are addressed, it is important that threshold shifts are also considered.

While it is difficult to predict the actual temperatures and temperature changes insects will experience in nature, and what treatments are ecologically relevant, our results highlight that using the same hardening treatments to compare plasticity across species that are adapted to different climates, or have different levels of tolerance, may underestimate plasticity in some species. Future studies that combine acclimation and hardening responses across several conditions, and link shifts in phenotypes to the induction of candidate genes across different species may help us understand the mechanisms underpinning threshold shifts and whether changes in plasticity are dictated by upper limits. Although this study focused on how threshold shifts and changes in plasticity at different developmental temperatures influence trade-off patterns across species, these issues also have implications for trade-off plasticity patterns in experimental evolution/selection experiments and biogeographical comparisons of plasticity across species or populations more broadly. We encourage future studies to reconsider the treatments they use to estimate and compare plasticity.

## Data Availability

The data are provided in electronic supplementary material [80].
